# Maternal and perinatal outcomes in pregnant women with rheumatic diseases treated with biosimilar TNF inhibitors

**DOI:** 10.1093/rap/rkae044

**Published:** 2024-03-19

**Authors:** Ana Teodósio Chícharo, Ana R Lopes, Sofia Barreira, Luísa Pinto, Ana R Cruz-Machado, Susana Capela

**Affiliations:** Rheumatology and Metabolic Bone Diseases Department, Hospital de Santa Maria, Centro Hospitalar Universitário Lisboa Norte, Lisbon Academic Medical Center, European Reference Network on Rare Connective Tissue and Musculoskeletal Diseases Network (ERN-ReCONNET) and RITA Full Member, Lisbon, Portugal; Rheumatology Department, Centro Hospitalar Universitário do Algarve, Hospital de Faro, Faro, Portugal; Rheumatology and Metabolic Bone Diseases Department, Hospital de Santa Maria, Centro Hospitalar Universitário Lisboa Norte, Lisbon Academic Medical Center, European Reference Network on Rare Connective Tissue and Musculoskeletal Diseases Network (ERN-ReCONNET) and RITA Full Member, Lisbon, Portugal; Instituto de Medicina Molecular João Lobo Antunes, Faculdade de Medicina da Universidade de Lisboa, Lisbon, Portugal; Rheumatology and Metabolic Bone Diseases Department, Hospital de Santa Maria, Centro Hospitalar Universitário Lisboa Norte, Lisbon Academic Medical Center, European Reference Network on Rare Connective Tissue and Musculoskeletal Diseases Network (ERN-ReCONNET) and RITA Full Member, Lisbon, Portugal; Instituto de Medicina Molecular João Lobo Antunes, Faculdade de Medicina da Universidade de Lisboa, Lisbon, Portugal; Department of Obstetrics, Gynecology and Reproductive Medicine, Hospital de Santa Maria, Centro Hospitalar Universitário Lisboa Norte, Lisbon, Portugal; Rheumatology and Metabolic Bone Diseases Department, Hospital de Santa Maria, Centro Hospitalar Universitário Lisboa Norte, Lisbon Academic Medical Center, European Reference Network on Rare Connective Tissue and Musculoskeletal Diseases Network (ERN-ReCONNET) and RITA Full Member, Lisbon, Portugal; Instituto de Medicina Molecular João Lobo Antunes, Faculdade de Medicina da Universidade de Lisboa, Lisbon, Portugal; Rheumatology and Metabolic Bone Diseases Department, Hospital de Santa Maria, Centro Hospitalar Universitário Lisboa Norte, Lisbon Academic Medical Center, European Reference Network on Rare Connective Tissue and Musculoskeletal Diseases Network (ERN-ReCONNET) and RITA Full Member, Lisbon, Portugal; Instituto de Medicina Molecular João Lobo Antunes, Faculdade de Medicina da Universidade de Lisboa, Lisbon, Portugal

Key message

- No safety or efficacy issues arose from using biosimilar TNFi during conception or pregnancy.

Dear Editor, Tumor necrosis factor (TNF) belongs to a group of cytokines that play a central role in the pathogenesis of several inflammatory conditions. TNF inhibitors (TNFi) work by neutralizing soluble TNF-α and preventing its binding to TNF receptors. This way, TNFi have become important treatment option for chronic inflammatory diseases, including rheumatoid arthritis (RA), psoriatic arthritis (PsA) and axial spondyloartrhitis (axSpA). Although these drugs cross the placenta (with the exception of certolizumab), safety data consistently demonstrated that TNFi exposure during pregnancy is not associated with adverse pregnancy outcomes (APO), congenital abnormalities or negative impact on postnatal development in infants [1]. The use of TNFi has been linked to a heightened risk of maternal, but not infantile, infections [2]. International guidelines support the continuation of TNFi throughout pregnancy to maintain maternal disease control. If sustained remission, they consider discontinuation in the third trimester to minimize the transfer of the drug to the infant, enabling the newborn to follow a regular vaccination schedule [3–5].

Biosimilar TNFi have highly similar characteristics to original TNFi and have shown clinically equivalent effectiveness and safety when compared with originator drugs. They are also more affordable, making treatment accessible to a wider range of patients. There is scarce data about their use in pregnancy and for this reason, they are not addressed in the latest guidelines on reproductive health for rheumatic patients. To date, there is only one observational retrospective study describing the outcomes of pregnant women exposed to biosimilar TNFi, with the diagnoses of not only rheumatic conditions (*N* = 9) but also inflammatory bowel disease (*N* = 9) [6].

Acknowledging the lack of data in this field, we performed an observational retrospective study of rheumatic patients exposed to biosimilar TNFi during conception, pregnancy and postpartum period. Patients were followed from 2018 until 2023 at a high-risk rheumatology-obstetric clinic of a tertiary hospital. Our aim was to describe maternal and perinatal outcomes in women using biosimilar TNFi and to assess the safety and efficacy of these drugs. All patients provided their written informed consent.

Five pregnancies in three Caucasian women were included, with the diagnoses of RA, axSpA and PsA. Table 1 summarizes maternal and perinatal outcomes and relevant clinical data. At the time of conception, all patients were in remission under biosimilar TNFi: one on infliximab (IFXb) and two on etanercept (ETAb). After a shared decision with the patients, the switch to certolizumab was not considered due to sustained remission under these biosimilars.

**Table 1.** Maternal and perinatal outcomes in women with rheumatic diseases treated with biosimilar TNF inhibitors during conception and pregnancy

Patient		Age at conception^a^	Obstetric history	Diagnosis	Disease duration^b^	DMARD at conception and during pregnancy (with dosing scheme)	Biosimilar latest administration^c^	Flare in pregnancy	Flare in postpartum	Maternal infections	Gestational age at delivery^c^	Birth weight (g)	Breastfeeding	Timing of biosimilar reinitiation	Newborn infections	Adverse pregnancy outcomes
A	Pregnancy 1	35	G1	Axial spondylo- arthritis	12	IFXb 5 mg/kg every 9 weeks	18	No	No^e^	No	39 + 3	3160	Yes^e^	28	No	No
	Pregnancy 2	38	G2P1		15	IFXb 5 mg/kg every 9 weeks	24	Yes—Sacroiliitis at 18^th^ WG		No	40 + 1	2990		16	No	No
B	Pregnancy 3	29	G1	Rheumatoid arthritis	8	SSZ 1000 mg/day and ETAb 50 mg (weekly before conception, every 2 weeks during pregnancy)	7	No		No	N/A	N/A		N/A	N/A	Miscarriage at 8 WG
	Pregnancy 4	30	G2P0A1		9		30	No		No	40 + 2	2915		4	No	No
C	Pregnancy 5	32	G3P0A2 (2 previous voluntary terminations of pregnancy)	Psoriatic arthritis	7	ETAb 50 mg weekly	26	No		Yes—urinary tract infection at 26^th^ WG	37 + 5	2982		6	Yes—urinary tract infection at 9 days old	No

a In years.

b In years before conception.

c In weeks of gestation + days.

d In weeks postpartum.

e N/A for patient B pregnancy 3.

DMARDs: Disease-modifying antirheumatic drugs; IFXb: infliximab biosimilar; ETAb: etanercept biosimilar; SSZ: sulfasalazine; WG: weeks of gestation; N/A: not applicable.

The first case refers to a nulliparous woman aged 35, diagnosed with axSpA HLAB27+ with axial and peripheral involvement and previous uveitis. At the time of conception, she was in remission under IFXb 5 mg/kg every 9 weeks, which she continued until 18 weeks of gestation (WG). At 39 WG, she delivered a healthy baby girl weighing 3160 grams. IFXb was resumed 7 months later, as the patient decided not to restart it during breastfeeding, as she maintained low disease activity. No APO or flares were noticed. Three years later, during a subsequent pregnancy, the patient remained on IFXb monotherapy at the same dosing scheme. However, she experienced a sacroiliitis flare at 18 WG, which resolved after a joint injection with methylprednisolone. IFXb was last administered at 24 WG, and the remaining pregnancy was uneventful. A healthy baby weighing 2990 g was born at 40 WG. The biosimilar was resumed four months postpartum.

The second patient was a 30-year-old woman diagnosed with seropositive RA, treated with ETAb 50 mg weekly, sulfasalazine 1 g/day and prednisolone (≤5mg/day). After conception, ETAb was adjusted to 50 mg every other week due to sustained remission, until 30 WG. The pregnancy progressed without any RA flares. At 40 WG, she delivered a healthy neonate weighing 2915 g. ETAb was restarted one month postpartum. No APOs were recorded. This woman, with negative antiphospholipid antibodies, had experienced a miscarriage at 8 WG the year before, while on remission and under the above-mentioned treatment.

The last case involves a 32-year-old woman with PsA with dominant peripheral involvement, under ETAb 50 mg weekly in monotherapy. ETAb was continued up to the 26^th^ WG, when it was stopped due to a *Streptococcus agalactiae* mild urinary tract infection (UTI), successfully treated with a course of antibiotics. Although the arthritis remained controlled, psoriasis relapsed after discontinuing the biosimilar, requiring the use of topical emollients. She gave birth to a healthy baby weighing 2989 g at 37 WG. However, at 9 days old, the newborn, who had no congenital urinary tract abnormalities, developed an urosepsis to *Escherichia coli*, requiring hospitalization and antibiotic intravenous therapy for 10 days. The mother restarted ETAb six weeks postpartum.

In conclusion, no new safety or efficacy issues arose from the use of IFXb or ETAb during conception, pregnancy or breastfeeding. Our data are reassuring and in line with studies of originator and biosimilar TNFi [6–8]. Patients under biosimilar TNFi should continue their usual therapy during conception and pregnancy to reduce the risk of flares and APO. TNFi biosimilars represent a more affordable treatment option that should be considered as standard practice in the near future. We acknowledge some limitations of our study, namely the retrospective design and the small sample size. Future studies with larger samples and prospective designs are needed to validate these findings and provide robust evidence.

## Data Availability

Data are available upon request.
